# Video-based pelvic floor muscle therapy for patients with pelvic floor disorders: A Protocol for a prospective single-arm pilot and feasibility study

**DOI:** 10.1371/journal.pone.0329883

**Published:** 2025-10-24

**Authors:** Samantha M. Linhares, Madeline L. D’Aquila, Kurt S. Schultz, Anne K. Mongiu

**Affiliations:** Division of Colon & Rectal Surgery, Department of Surgery, Yale School of Medicine, New Haven, Connecticut, United States of America; Iran University of Medical Sciences, IRAN, ISLAMIC REPUBLIC OF

## Abstract

The number of patients who suffer from pelvic floor disorders increases with age and can have a significant impact on quality of life. The first-line treatment for these different disorders includes pelvic floor rehabilitation. However, there are high rates of non-compliance with completing the recommended duration of treatment due to delays in appointments and time constraints. Thus, the primary goal of this study is to evaluate the feasibility and acceptability of an online eight-week video-based pelvic floor muscle therapy program. A secondary goal is determining whether the treatment can improve quality of life and symptoms. This study is a registry-based pilot single-arm prospective trial (NCT06689891: Video-Based Pelvic Floor Muscle Therapy). A single-arm design was utilized because our primary aim was to assess feasibility rather than comparative efficacy. Methodical guidance recommends a single-arm approach when outcomes focus on process measures instead of hypothesis testing. This design allows us to evaluate whether the intervention can be delivered as intended, establish feasibility before a larger trial, and maintain flexibility for modification without compromising a control group. Eligible participants will be offered the online program as an alternative to in-person pelvic floor rehabilitation. Primary timepoints include a pre-intervention in-person visit with a licensed pelvic floor therapist and the 8-week video-based pelvic floor muscle therapy program. There will be a midpoint evaluation followed by a post-intervention visit with the same pelvic floor therapist, where participants will be graded on their ability to complete the various exercises to assess efficacy. A survey assessing the online-based program’s usability will be conducted post-intervention. Patient-reported outcome measures, including quality of life and symptom changes, will be collected pre-, mid-, and post-intervention. As this is a pilot trial, the goal is to establish the acceptability and feasibility of a video-based pelvic floor muscle therapy program as an alternative to in-person treatment.

## Introduction

Pelvic floor disorders, which include pelvic organ prolapse, urinary incontinence, and anorectal dysfunction, affect up to 25–32% of women during their lifetime.[1,2] These disorders can be debilitating and will affect quality of life (QoL) for up to 50% of patients.[3] Pelvic floor rehabilitation (PFR) is the first-line treatment for patients with pelvic floor disorders and is part of the management algorithm for other disease processes, such as low anterior resection syndrome (LARS).[4–6] The exercises commonly used in PFR include pelvic floor muscle therapy (PFMT) with biofeedback training, rectal balloon training, or electrical stimulation.[7–9] Together, these techniques improve the strength, tone and endurance of the pelvic floor musculature and sphincter complex.[8] They have decreased symptoms ranging between 50–80% for fecal incontinence.[10,11] A meta-analysis of 18 trials utilizing PFMT for urinary incontinence found an improvement in symptoms in all studies.[12]

Despite PFMT being shown to be efficacious for numerous different pelvic floor dysfunction disorders, less than 50% of patients referred are compliant with the recommended duration of treatment.[13,14] A study of 180 patients referred for PFR found that 34% of patients did not attend a single session, and only 29% completed the entire duration.[15] Previous studies found that inconvenience, cost, lack of knowledge, and discomfort around the concept of PFMT are all reasons why patients may have poor compliance.[16–18] In addition, a survey study our lab conducted showed that the waiting period once referred for PFMT is nine weeks due to the limited number of available pelvic floor therapists. Patients have shown a positive reception to telehealth-based PFMT visits with recent studies showing high compliance rates and satisfaction when compared to in-person visits. However, telehealth visits are still limited based on pelvic floor therapists availability which continues to be a major limiting factor.[19–22] It is unknown if a hybrid approach to PFMT, including both in-person and self-directed video-based training, may help alleviate these barriers while providing the same adequate amount of guidance as well as improvement in symptoms and in life satisfaction for patients. Thus, this study aims to evaluate the feasibility and acceptability of an online eight-week video-based PFMT for pelvic floor disorders. We hypothesize that an online video-based PFMT program is a feasible alternative to in-person pelvic floor muscle therapy for patients with pelvic floor disorders.

## Materials and methods

**Fig 1:**
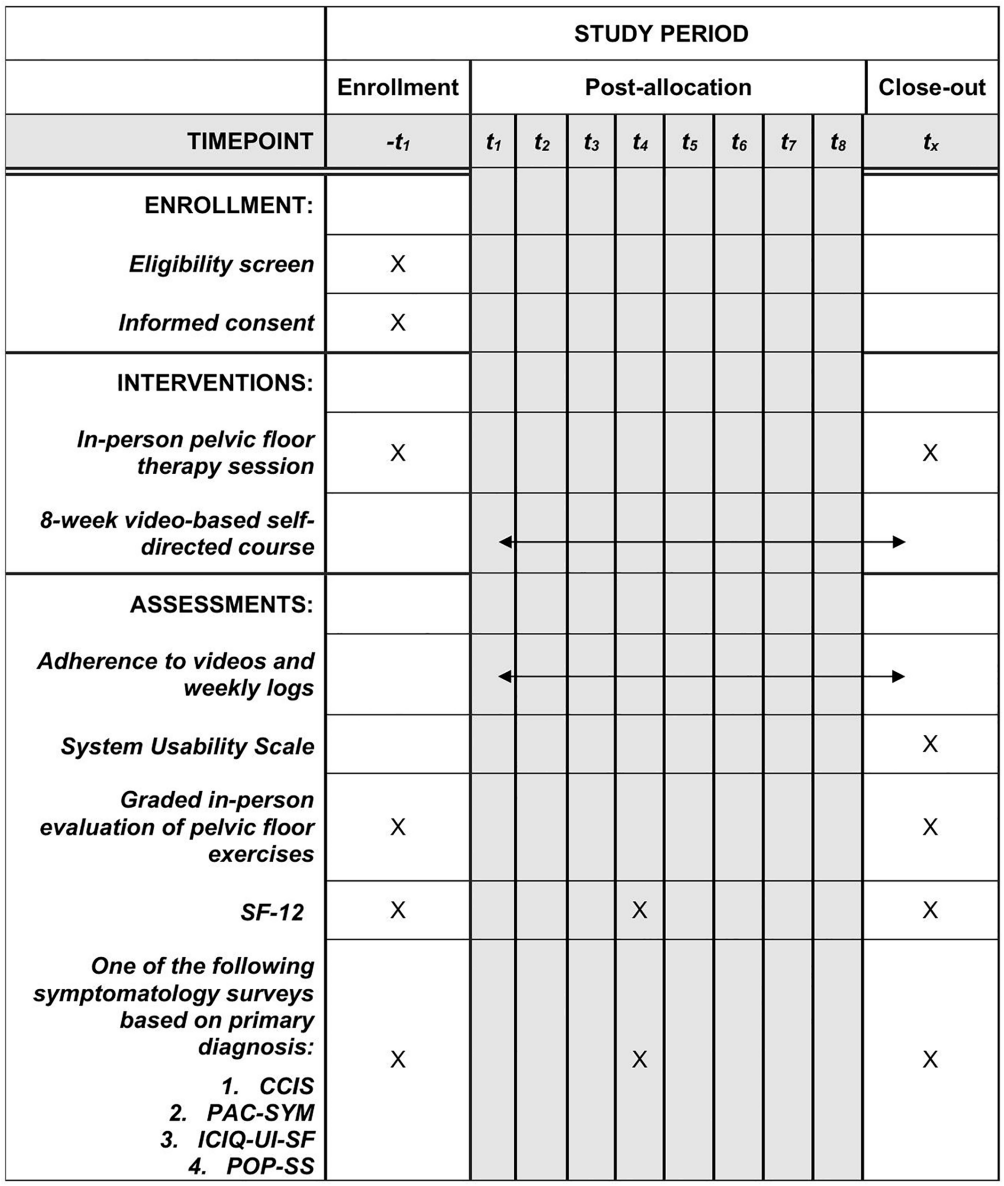
SPIRIT schedule of enrollment, interventions, and assessments.

This study is a prospective, registry-based, single-arm trial assessing feasibility and acceptability of an online eight-week video-based PFMT for pelvic floor disorders (NCT06689891: Video-Based Pelvic Floor Muscle Therapy). [23] The protocol has received approval from the Yale University Institutional Review Board (#2000037163). This study aims to evaluate the feasibility and acceptability of an online video-based PFMT program, a novel delivery model for a proven first-line treatment for pelvic floor disorders (S2 Protocol in S1 File). This will be a hybrid design with remote video-based PFMT and two in-person pelvic floor therapy sessions at a tertiary academic hospital in the United States. The schedule of enrollment, interventions, and assessments is shown in Fig 1. Study enrollment began on June 6, 2025. Enrollment is ongoing, and the recruitment period has not yet ended. We estimate participant recruitment will be completed by December 2025. Data collection will be completed by March 2026. We expect to have preliminary results by April 2026. The protocol is reported according to the clinical trial SPIRIT checklist guidelines (S1 Checklist).

### Sample size

As this is a pilot study, we will aim to recruit 20 adult participants, which is within the range typical of other pilot studies to test feasibility and acceptability. Based on prior studies and guidance for feasibility tests, we did not conduct a power calculation for sample size in line with the standard for feasibility studies. [24,25] Efficacy results from this pilot study will be used for sample size calculations in future trials. Study sites include outpatient urogynecology clinic and colorectal surgery clinic. Twenty English-speaking adult females at least 18 years of age with a diagnosis of urinary incontinence, pelvic organ prolapse, pelvic pain, fecal incontinence, or constipation will be recruited during their urogynecology or colorectal surgery clinic visit for one of these conditions (Table 1). Exclusion criteria include non-female sex, participants who cannot speak or read English, those who do not have reliable access to the internet, and those who do not know how to navigate the internet. Researchers will recruit participants through follow-up emails or phone calls to participants who have expressed initial interest in the study to their primary healthcare provider.

**Table 1:** Inclusion and exclusion criteria.

Inclusion Criteria	Exclusion Criteria
Age > 18	Male sex
Diagnosis of pelvic floor disorder	Non-English speakers
Ability to perform pelvic floor muscle therapy	Unable to access web-based videos
Reliable internet access	Limited internet proficiency

### Intervention

**Fig 2:**
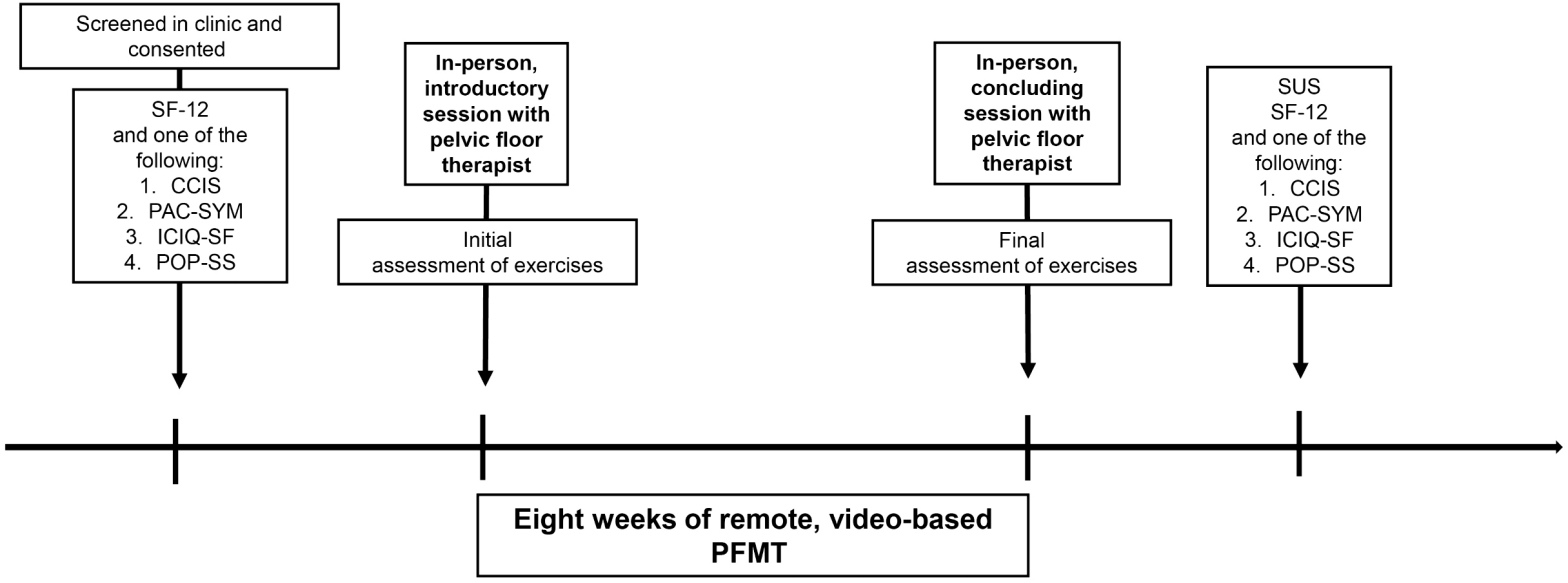
Study design for a proposed feasibility trial of using video-based pelvic floor muscle therapy (PFMT) intervention for participants. At the initial clinic visit where participants are eligible to receive PFMT, they will be offered to enroll in video-based PFMT. If they are consented, they will asked to complete the Short Form Health Survey (SF-12), and one of the four following symptom-specific surveys: 1) Cleveland Clinic Incontinence Score (CCIS), 2) Patient Assessment of Constipation Symptoms (PAC-SYM), 3) International Consensus on Incontinence-Urinary Incontinence Questionnaire Short Form (ICIQ-UI-SF) or 4) Pelvic Organ Prolapse Symptom Score (POP-SS) which they will complete again after the 8 weeks of video-based PFPT. After conclusion of the eight weeks, they will also complete the System Usability Survey (SUS) to evaluate for ease of use of the web-based application. They will meet with a pelvic floor therapist prior to initiation of the video-based PFMT and again at the conclusion with an assessment of exercise competency preoperatively and postoperatively.

The baseline study visit will be a standard of care visit with an in-person pelvic floor therapist. This visit will introduce the concepts important to pelvic floor exercises and assess the participant’s baseline function with graded evaluation of a standard set of pelvic floor exercises, which is discussed further in the secondary outcome of efficacy below. Participants will then complete eight weeks of online video-based PFMT. The duration of eight weeks was chosen based on the average duration reported in the literature and based on expert opinion from our pelvic floor physical therapists. [26,27] The videos were professionally recorded and developed by our institution in collaboration between pelvic floor physical therapists and the Department of Colon and Rectal Surgery. These videos are not currently available to researchers or clinicians outside of our health system. We primarily worked with one physical therapist who has been practicing general physical therapy for over 20 years and pelvic floor physical therapy for five years. The videos have four sections: introduction followed by beginner, intermediate, and advanced exercise demonstrations. The videos were designed by our pelvic floor physical therapists based on knowledge of progression of difficulty of exercises and reflect the exercises that the therapists administer to patients in-person. The progression of these videos is released after a set amount of time. Each section includes a 30-minute video. Participants are recommended to engage with the program for 20–25 minutes at least three days a week. Participants will be sent a weekly log to self-report the number of days they exercised (S3 Weekly Log). The weekly logs are electronically messaged to participants, and participant responses are self-reported. There will be mid-point feedback at 4 weeks, where participants repeat the initial surveys to assess life satisfaction and symptomatology. After the video-based PFMT program is completed, participants will have a second in-person session with the same physical therapist for repeat graded assessment of the PFMT exercises. They will receive a $50 gift card upon completion of the second in-person session. We have opted to have in-person visits pre and post intervention to establish rapport with participants, as a prior qualitative review discussed how patients with solely telehealth interventions miss the social connection. In addition, it allows insight into efficacy of the intervention based on expert evaluation and to garner additional feedback from participants about their experience with the program. [28] Although challenges to in-person visits exist, such as transportation difficulties, we opted for a hybrid approach to evaluate efficacy with repeated in-person assessments. Participants will also complete the System Usability Scale (SUS) and appropriate symptomatology survey post-intervention, ideally within two weeks of the final in-person visit (Fig 2).

### Relevant concomitant care or prevention

During this study period, participants will be asked to abstain from participating in additional supervised in-person pelvic floor therapy sessions. Participants may choose at any point to opt out of the study and pursue the standard of care with in-person supervised pelvic floor therapy for participant preference or worsening symptoms. Participant symptomatology and QoL will be assessed at the midpoint feedback and participants will be notified if their symptoms are improved or worsened. They will then be offered the opportunity to complete additional recommended number of sessions in person.

### Primary outcomes

#### Feasibility.

The primary outcome measure for feasibility is participant adherence to the eight-week online video-based PFMT program. Exercise adherence will be determined by self-reported weekly logs of exercise frequency. Adherence will be calculated as the number of days exercised divided by the total number of days in the study period (i.e., 56 days). We define adherence as exercising at least three days per week. We aim for participants to have at least 80% adherence to this exercise goal, as is common among exercise or physical activity intervention studies [29–31]. To account for noncompliance and attrition, all days in the study period will be included in the denominator, regardless of whether the participant performed the exercise or completed the study. Attrition will be measured by number of participants who did not complete the eight-week study divided by the number of participants who did complete the study. The online video-based program will be digitally monitored by research staff. Video adherence will be calculated as the total number of videos viewed divided the total number of videos included in the course.

#### Acceptability.

The SUS is the primary outcome measure for acceptability. This 10-item questionnaire was initially developed in 1986 to capture a user’s evaluation of an online system’s usability with a 10-item questionnaire. [32,33] It is the most widely used questionnaire to evaluate health technology interfaces and has robust data for validation. [34,35] The SUS is administered at the completion of the study to determine the ease of independently following the videos and exercises.

### Secondary outcomes

#### Efficacy.

The outcome measure for efficacy is a pelvic floor therapist’s graded evaluation of exercises. The graded assessment is based on evaluating a set of standard of care pelvic floor exercises, ranging from easy to more difficult, as has been used in other pelvic floor therapy studies. [36] Each exercise will be given a score of one to three, with one indicating that the participant is unable to complete the exercise without assistance, two indicating the participant can partially complete the exercise independently, and three indicating the participant can complete the exercise independently. A total of five exercises of varying difficulty will be assessed.

#### Life satisfaction.

Life satisfaction changes are assessed with the Short Form-12 (SF-12), along with changes in symptomatology based on one of the four appropriate symptom-focused surveys: 1) Cleveland Clinic Incontinence Score (CCIS), 2) Patient Assessment of Constipation Symptoms (PAC-SYM), 3) International Consultation on Incontinence-Urinary Incontinence Questionnaire Short Form (ICIQ-UI-SF), and 4) Pelvic Organ Prolapse Symptom Score (POP-SS).

The SF-12 is a 12-item questionnaire to assess quality of life in the physical and mental domains. It is an abbreviated form of the longer SF-36, which is a 36-item questionnaire. The SF-12 has been tested with good reliability and validity compared to the SF-36, and we have opted to use this shorter survey to reduce the burden on participants.[37] The CCIS is one of the most common instruments for assessing fecal incontinence.[38] This is a five-item questionnaire designed to assess patient symptomatology related to incontinence in terms of type of leakage (i.e., solid versus liquid versus flatus), the necessity to wear hygiene pads, and the impact on lifestyle. Compared to several commonly used incontinence scales, this questionnaire is equally validated and more succinct. [39] The PAC-SYM is a 12-item questionnaire developed using patient perspectives to assess constipation on three symptom subscales: abdominal, rectal, and stool. [40] It has been widely validated and is one of the most common assessments for constipation. [41] The ICIQ-UI-SF is a four-item questionnaire that assesses the frequency, severity, and impact on QoL of urinary incontinence and is the most commonly used questionnaire worldwide [42] and is endorsed by multiple societies, including the American Society of Colon and Rectal Surgeons and the American Urogynecologic Society. [41] The POP-SS is a seven-item questionnaire developed to assess the severity of pelvic organ prolapse symptoms and has been extensively validated. [43,44] (Table 2)

**Table 2:** List of primary and secondary outcomes.

	Variable of Interest (VOI)	Description	Measurement Properties	Rationale
**Primary Outcome**	Adherence to exercises	Compliance with recommended number of weekly exercises (3x a week for 20–25 minutes)	Weekly log percentage	Feasibility most assessed by adherence to the intended treatment with a goal of minimum 80% compliance
	System usability scale (SUS) [32]	10-item questionnaire used to assess usability of a system	Reliable (Cronbach’s alpha 0.7–0.95) [34]	Most widely used scale to assess various system usability to assess acceptability
**Secondary Outcome**	Graded evaluation by pelvic floor therapist	Graded scaling of 1–3 for each pelvic floor exercise as evaluated by a pelvic floor therapist	Scoring a total of five exercises with maximum score of 15	Commonly used method of exercise evaluation for pelvic floor therapy and other physical activity
	Short-Form 12 (SF-12) [37]	12-item questionnaire to assess quality of life	Reliable with R^2 = 0.905 (physical) and 0.938 (mental) compared with SF-36. Relative validity median = 0.67 (physical) and 0.97 (mental) compared to SF-36 [37]	Shortened version of SF-36 but proven to be equally valid at assessing patient QoL in both physical and mental domains
	Cleveland Clinic Incontinence Score (CCIS) [38]	5-item questionnaire to assess types of leakage (solid/liquid/gas), necessity to wear hygiene pads, and lifestyle changes	Reliable (ICC = 0.86–0.92) [38]	Most used tool for incontinence; in comparison to other equally validated questionnaires is more succinct
	Patient Assessment of Constipation Symptoms (PAC-SYM) [40]	12-item questionnaire with 3 symptom scales: abdominal, rectal and stool to assess constipation symptoms	Reliable (Cronbach’s alpha = 0.89; ICC = 0.75) [40]	Widely used assessment of constipation
	International Consultation on Incontinence Questionnaire-Urinary Incontinence Short Form (ICIQ-UI-SF) [42]	4-item questionnaire to assess frequency, severity, and impact on QoL of urinary incontinence	Reliable (Cronbach’s alpha = 0.85–0.95, ICC = 0.84, MCID = 4 points) [45]	Commonly used questionnaire developed by expert committee
	Pelvic Organ Prolapse Symptoms Score (POP-SS) [44]	7-item questionnaire to assess severity of pelvic organ prolapse symptoms	Reliable (Cronbach’s alpha = 0.823, MCID = 1.5 points) [43]	Widely used instrument to assess pelvic organ prolapse symptoms

### Statistical analysis

As this is a feasibility study, the primary analyses will be descriptive including the retention rate of participants, the percentage of videos completed, and adherence to the recommended weekly number of exercises. Participant demographics and clinical characteristics, including age, sex, race, ethnicity, and indication for pelvic floor referral, will be summarized as descriptive statistics. The primary outcome of feasibility will be defined as the number of participants meeting the minimum of 80% adherence to the program. Exercise adherence will be calculated as the number of days exercised divided by the total number of days in the study period (i.e., 56 days). Video adherence will be calculated as the total number of videos viewed divided the total number of videos included in the course.

Acceptability will be evaluated using the SUS scores post-intervention. For the secondary outcome of efficacy, we will report mean differences, 95% confidence intervals, Cohen’s d, and p-values between the pre and post intervention for the graded exercise evaluation scores by the pelvic floor therapist. For the secondary outcome of life satisfaction, we will also report mean differences, 95% confidence intervals, Cohen’s d, and p-values at the three different time points (pre-intervention, midpoint, and post-intervention). We will use a longitudinal data analysis approach to evaluate survey responses over time. We will perform either a paired t-test or Wilcoxon Signed Rank Test depending on parameter testing. We will also fit a linear mixed effects model. Participants with missing data related to secondary outcomes will be excluded from this analysis as imputation methods would be unstable given the small sample size. The extent and reasons for missingness will be reported. Analyses will be descriptive and hypothesis-generating only, as the study is not powered for statistical inference.

### Data management plan

The survey information will be either researcher-administered or independently completed by the study participant using an institution-licensed REDCap platform (https://redcapynh-p11.ynhh.org/). If there are technical difficulties, a paper-based version of the assessment tool will be substituted and inputted into the system later. Demographic and clinical data will be extracted from the electronic medical records (e.g., Epic® chart reviews) and will be securely stored in the HIPAA-compliant REDCap database. The assessment by the pelvic floor therapist will also be securely input into the REDCap database. The full protocol, datasets, and statistical code used during this study will be made available from the corresponding author on reasonable request.

### Monitoring

The study is deemed minimal risk as participants can perform on their own and would not increase the magnitude of harm or discomfort than those ordinarily encountered in routine physical exams. The exercises as part of the program are not outside the scope of the standard of care. No interim analyses are planned for this study because the intervention is low-risk, and the primary purpose of this pilot study is feasibility not efficacy.

Oversight of trial safety and data integrity will be by the study team and internal institutional monitoring mechanisms at least monthly. In the unlikely event that an adverse event occurs, a written report will be submitted within five calendar days of the Principal Investigator becoming aware of the event to the IRB and appropriate funding and regulatory agencies. The study team and sponsor will review the data and determine if modification or early termination is warranted.

### Ethics and dissemination

The protocol has received approval from the Yale University Institutional Review Board (#2000037163). Authorized research study staff will obtain written informed consent from participants prior to starting the study. Important protocol changes will be communicated to participants, study staff, and the IRB by the study investigator.

Data will be securely stored in an institutional HIPPA protected online database with a built-in auditing system. Only authorized study staff will have access to the data. Study results will be submitted for presentation and publication at future national meetings and in surgical journals. Authorship criteria will be defined by the International Committee of Medical Journal Editors. Study investigators do not have any conflicts of interest to declare.

## Discussion

This study protocol is designed to test the feasibility and acceptability of a web-accessible online eight-week PFMT program as an alternative delivery method to the standard of care for the treatment of PFD. PFD is a constellation of disorders that affects nearly one-third of the female population, with a notable impact on QoL.[3] Despite PFMT being a first-line treatment for these disorders, many barriers are in place that prohibits patients from completing the recommended number of sessions or even attending one session. It is not known whether a hybrid, online video-based PFMT program would be a feasible and safe alternative to in-person PFR.

Potential pitfalls to this project include a small sample size with multiple indications for PFR, which may increase variability in determining acceptability. To minimize this, we will group results into two broad indications: urogynecology and colorectal. Another pitfall is potential loss to follow-up and lack of adherence during the self-directed portion. We have a $50 financial incentive for participants after the final in-person assessment, which could introduce response bias and attrition bias as has been shown in previous studies. [46,47] We have attempted to mitigate these biases by having the payment at the end of the study with an amount that compensates for the participants’ time and travel for in-person appointments but does not exceed this. We will also provide weekly log check-ins to encourage participants to continue exercising.

The results of this study have the potential to be transformative for individuals referred for PFR by establishing a novel healthcare delivery model for the treatment of pelvic floor disorders. This pilot feasibility trial will provide preliminary data for a larger, multicenter study to evaluate the use of this delivery model as a potential alternative or supplement to in-person pelvic floor therapy. The results will help to assess if the video-based curriculum is easily understood and potentially remove the need for an in-person pre and post intervention assessment, as this remains a major barrier. It will also provide preliminary information about potentially which pelvic floor disorders are best treated with the current curriculum and provide insight into how modifications may be needed for certain disorders. It will also allow for feedback on the video-delivery platform to ensure that it can be easily accessible if scaled for a larger study.

There are limitations to scaling this video-based PFMT program. Currently, the videos are currently only available in English, which excludes non-English speaking patients. In addition, patients need to have adequate digital literacy to navigate the website, and this may limit the utility of a virtual format. On the other hand, participants who are socioeconomically disadvantaged, such as participants with transportation needs, might benefit from this more accessible version of pelvic floor physical therapy. [48] Next steps include creating non-English versions of the video platform and directly integrating the videos into electronic medical record patient portals. Further directions include adoption of this online video-based platform for other diseases and conditions, such as adapting successful prehabilitation programs to an online delivery model for patients prior to surgery. [49,50] Online alternatives continue to be on the rise to provide patients with additional flexibility in an increasingly busy world. Development of a web-accessible video-based PFMT program would target an unmet need, benefit individuals suffering from a variety of PFD, and pave the way for other alternatives in care.

## Supporting information

S1 Checklist SPIRIT Checklist. (PDF)

S1 File Study Protocol. Clinical study protocol approved by the institutional review board.
(PDF)

S2 File Weekly Log. Weekly logs provided to study participants for self-reporting of weekly exercise frequency.
(PDF)
