# Supplementary material for: Video-based pelvic floor muscle therapy for patients with pelvic floor disorders: A Protocol for a prospective single-arm pilot and feasibility study
Source: PLoS One. 2025 Oct 24;20(10):e0329883. doi: 10.1371/journal.pone.0329883 (PMC12551819; doi:10.1371/journal.pone.0329883)
Supplement: S1 File — Clinical study protocol approved by the institutional review board. (PDF) [file pone.0329883.s002.pdf]

CLINICAL STUDY PROTOCOL

Observational Study of Individual or Group Template

Utilizing Remote, Video-Based Pelvic Floor Physical Therapy: A Feasibility Trial

Protocol Number

2000037163

Protocol Version

April 5th 2025 Version 1.3

Confidentiality Statement:

Synopsis

**Purpose**<br>The goal of the proposed study is to evaluate the feasibility and accessibility of pragmatic, remote, video-based pelvic floor muscle therapy (PFMT) for patients.
**Primary Objective**<br>The primary objective of this study is to determine the feasibility of use of a video-based PFMT.
**Secondary Objective**<br>The secondary objective is to determine whether PFMT can improve quality of life (QoL) and reduce symptom burden.
**Study Design**<br>Patients will complete a series of surveys before and after the intervention. They will complete 8 weeks of video-based PFMT with an initial and final in-person session with a pelvic floor therapist.
**Study Date Range and Duration**<br>December 2024 – August 2026
**Number of Study Sites**<br>Yale New Haven Hospital
**Primary Outcome Variables**<br>We will use direct patient evaluation by pelvic floor therapists, a validated system usability survey to evaluate the ease of use of the web-based platform containing PFMT videos and monitor for adverse events.
**Secondary and Exploratory Outcome Variables (if applicable)**<br>Short Form 12 score, Cleveland Clinic Incontinence Score (CCIS), Patient Assessment of Constipation Symptoms (PAC-SYM), International Consultation on Incontinence Questionnaire Short Form (ICIQ-SF), and Pelvic Organ Prolapse Symptom Score (POP-SS) to assess improvement in symptoms and quality of life.
**Study Population**<br>Study population will include patients who have an indication for referral for pelvic floor rehabilitation (PFR).
**Number of Participants**

We plan to enroll 10-20 patients. This is a pilot study aimed at assessing feasibility, so this sample size is similar to other feasibility trials.

Study Schedule

Patients will be expected to complete a 5–10-minute survey at initial enrollment. They will have one in-person session with a pelvic floor therapist prior to starting the 8-week video-based PFMT course. They will have a midpoint check in after 4 weeks to monitor progress. Afterwards, they will have a final in person session with a pelvic floor therapist along with a final set of surveys that should take an estimated 10-15 minutes. We have also attached a figure with the study design.

Protocol Revision History

Version Date	Summary of Substantial Changes
10/21/24	First submission
12/11/24	Second submission
3/7/25	Third submission
3/11/25	Fourth submission
3/13/25	Fifth submission

Statement of Compliance

This document is a protocol for a human research study. The purpose of this protocol is to ensure that this study is to be conducted according to the Common Rule at 45CFR46 (human subjects) and other applicable government regulations and Institutional research policies and procedures.

Abbreviations

Abbreviation	Explanation
**PFMT**	**Pelvic floor muscle therapy**
**PFR**	**Pelvic floor rehabilitation**
**QoL**	**Quality of life**

Glossary of Terms

Glossary	Explanation

Table of Contents

Preface 2

Synopsis 2

Purpose 2

Primary Objective 2

Secondary Objective 2

Study Design 2

Study Date Range and Duration 2

Number of Study Sites 2

Primary Outcome Variables 2

Secondary and Exploratory Outcome Variables (if applicable) 2

Number of Participants 2

Study Schedule 3

Protocol Revision History 4

Statement of Compliance 5

Abbreviations 6

Glossary of Terms 7

1 Background/Literature Review 11

1.1 Background 11

1.2 Prior Experience (if applicable) 12

2 Rationale/Significance 12

2.1 Rationale and Study Significance 12

2.2 Purpose of Study/Potential Impact 12

2.3 Potential Risks and Benefits 12

2.3.1 Potential Risks 12

2.3.2 Potential Benefits 13

3 Study Purpose and Objectives 13

3.1 Hypothesis 13

3.2 Primary Objective 13

3.3 Secondary Objective (if applicable) 13

4 Study Design 14

4.1.1 General Design Description 14
4.1.2 Study Date Range and Duration 14
4.1.3 Number of Study Sites 14
4.2 Outcome Variables 14
4.2.1 Primary Outcome Variables 14
4.2.2 Secondary and Exploratory Outcome Variables (if applicable) 14
4.3 Study Population 14
4.3.1 Number of Participants 14
4.3.2 Eligibility Criteria/Vulnerable Populations 14
5 Study Methods/Procedures 15
5.1 Study Procedures 15
5.1.1 Data Collection 15
5.1.2 Adverse Events Definition and Reporting 15
5.2 Study Schedule 16
5.3 Informed Consent 16
5.3.1 Screening (if applicable) 16
5.3.2 Recruitment, Enrollment and Retention (if applicable) 16
5.3.3 Study Visits (is applicable) 17
5.4 Statistical Method 17
5.4.1 Statistical Design 17
5.4.2 Sample Size Considerations 17
5.4.3 Planned Analyses 17
5.4.4 Analysis of Subject Characteristics (if applicable) 18
5.4.5 Interim Analysis (if applicable) 18
5.4.6 Handling of Missing Data 18
6 Trial Administration 18
6.1 Ethical Considerations: Informed Consent/Assent and HIPAA Authorization 18
6.2 Institutional Review Board (IRB) Review 18
6.3 Subject Confidentiality 18
6.4 Deviations/Unanticipated Problems 19
6.5 Data Quality Assurance 20

6.6 Study Records 20
6.7 Access to Source 20
6.8 Data or Specimen Storage/Security 21
6.9 Retention of Records 21
6.10 Study Monitoring 21
6.11 Study Modification...21
6.12 Study Completion..21
6.13 Funding Source...21
6.14 Conflict of Interest Policy...21
6.15 Publication Plan...21
Appendices 23
List of Tables..24

1 Background/Literature Review

1.1 Background

Pelvic floor rehabilitation (PFR) is a group of therapeutic techniques used to help patients with pelvic floor dysfunction, such as urinary incontinence or fecal incontinence. The exercises commonly use in PFR include pelvic floor muscle therapy (PFMT) with biofeedback training, rectal balloon training or electrical stimulation[1-3]. Together, these techniques aim to improve the strength, tone and endurance of the pelvic floor musculature and sphincter complex[2]. For fecal incontinence, they have shown to decrease symptoms ranging between 50-80%[4,5]. A meta-analysis looking at 18 trials utilizing PFMT for urinary incontinence found an improvement in symptoms in all studies[6].

Despite PFMT being shown to be efficacious for numerous different pelvic floor dysfunction disorders, less than 50% of patients referred are compliant with the recommend duration of treatment[7,8]. Previous studies found that inconvenience, cost, lack of knowledge and discomfort around the concept of PFMT are all reasons why patients may have poor compliance[9-11]. Patients have shown a positive reception to telehealth-based PFMT visits[12]. It is not known if a hybrid approach to PFMT including both in-person and video-based training videos may help to alleviate some of these barriers while providing the same adequate amount of guidance, improvement in symptoms and QoL for patients. Thus, the purpose of this study is to evaluate the feasibility and acceptability of a web-accessible 8-week video-based PFMT for pelvic floor disorders.

References:

1. Dulskas A, Smolskas E, Kildusiene I, Samalavicius NE. Treatment possibilities for low anterior resection syndrome: a review of the literature. *International journal of colorectal disease*. 2018;33:251-260.

2. Scott KM. Pelvic Floor Rehabilitation in the Treatment of Fecal Incontinence. *Clin Colon Rectal Surg*. 2014/09/24 2014;27(03):99-105. doi:10.1055/s-0034-1384662

3. van der Heijden J, Kalkdijk-Dijkstra A, Pierie J, et al. Pelvic floor rehabilitation after rectal cancer surgery: A multicenter randomized clinical trial (FORCE trial). LWW; 2022.

4. Damon H, Siproudhis L, Faucheron J-L, et al. Perineal retraining improves conservative treatment for faecal incontinence: a multicentre randomized study. *Digestive and Liver Disease*. 2014;46(3):237-242.

5. Norton C, Whitehead W, Bliss D, Harari D, Lang J. Management of fecal incontinence in adults. *Neurourology and Urodynamics: Official Journal of the International Continence Society*. 2010;29(1):199-206.

6. Dumoulin C, Hay-Smith J, Habée-Séguin GM, Mercier J. Pelvic floor muscle training versus no treatment, or inactive control treatments, for urinary incontinence in women: a short version Cochrane systematic review with meta-analysis. *Neurourology and urodynamics*. 2015;34(4):300-308.

7. Ross JH, Sinha A, Propst K, Ferrando CA. Adherence to Pelvic Floor Physical Therapy Referrals in Women With Fecal Incontinence. *Female Pelvic Med Reconstr Surg*. Mar 1 2022;28(3):e29-e33. doi:10.1097/spv.0000000000001140

8. Woodburn KL, Tran MC, Casas-Puig V, Ninivaggio CS, Ferrando CA. Compliance with pelvic floor physical therapy in patients diagnosed with high-tone pelvic floor disorders. *Urogynecology*. 2021;27(2):94-97.

9. Dumoulin C, Hay-Smith J, Frawley H, et al. 2014 consensus statement on improving pelvic floor muscle training adherence: International Continence Society 2011 State-of-the-Science Seminar. *Neurourology and urodynamics*. 2015;34(7):600-605.

10. Washington BB, Raker CA, Sung VW. Barriers to pelvic floor physical therapy utilization for treatment of female urinary incontinence. *American journal of obstetrics and gynecology*. 2011;205(2):152. e1-152. e9.

11. Zoorob D, Higgins M, Swan K, Cummings J, Dominguez S, Carey E. Barriers to pelvic floor physical therapy regarding treatment of high-tone pelvic floor dysfunction. *Urogynecology*. 2017;23(6):444-448.

12. ter Haar CM, Class QA, Kobak WH, Pandya LK. Telehealth in a Pelvic Floor Physical Therapy Clinic: A Retrospective Cohort Study. *Urogynecology*. 9900:10.1097/SPV.0000000000001510. doi:10.1097/spv.0000000000001510

1.2 Prior Experience (if applicable)

Our group conducted a study on patient reported barriers to completion of in-person PFR. We found the average time between referral and attendance of the first PFR session was 10 weeks. The two major barriers to completion included delay in available sessions (24%) and travel distance for attendance (20%). Furthermore, 35% of patients stated they would be amenable to a hybrid option using either telehealth or video-based strategies and 58% of patients would attend more sessions if there was a video-based option.

2 Rationale/Significance

2.1 Rationale and Study Significance

PFMT is a non-invasive tool to improve symptoms of pelvic floor dysfunction, but there are many barriers for patients to successfully attend and complete the trainings, with the most common reasons cited in a retrospective study conducted by our group including delay in available sessions and travel distance for attendance. It is not known if these exercises could be adequately learned through a video-based training module that would give patients back privacy and time.

2.2 Purpose of Study/Potential Impact

Evaluating the feasibility and safety of video-based PFMT will allow us to determine if this video-based training could be offered as a treatment alternative for patients with pelvic floor dysfunction.

2.3 Potential Risks and Benefits

2.3.1 Potential Risks

Pelvic Floor Muscle Therapy Sessions (in-person);

Participating in PFMT can be time consuming and may be uncomfortable for some patients. There is no guarantee that the instruction and exercises in these sessions will prevent patients from having persistent or worsening pelvic floor dysfunction. A trained physical therapist will be supervising and teaching these sessions. Patients can opt out of certain exercises if desired.

Pelvic Floor Muscle Therapy Sessions (video-based):
Practicing PFMT using the video-based application may similarly be time consuming and uncomfortable for patients to perform. Additionally, as patients will be performing these exercises alone without supervision, there is the risk of harm to self. There is no guarantee that the instruction and exercises in these sessions will prevent patients from having persistent or worsening pelvic floor dysfunction as they are opting out of standard of care visits. Patients can choose to opt out of certain exercises if desired.

Surveys:
The surveys administered may be tiring and/or sensitive for patients. All patients will be informed that any questions they do not feel comfortable answering are optional. The surveys used to evaluate for fecal incontinence, constipation and QoL are all validated questionnaires from pre-existing literature. The survey used to evaluate the efficacy of the video-based PFMT is also validated and should not incur any harm to patients.

Medical Record Review:
The medical record of each patient will be reviewed by the study staff to extract demographic information. The risk of breach of confidentiality will be minimized with adherence to the Yale New Haven Health Data Security rules. The questionnaire and outcomes data will be stored on HIPAA compliant RedCap database.

We do not anticipate any long-range risks.

2.3.2 Potential Benefits

Short term: Patients may have improvement in pelvic floor dysfunction and QoL

Long term: Patients may have improvement in pelvic floor dysfunction and QoL. If successful, the videos could be developed into a mobile based application and offered as an alternative treatment method.

3 Study Purpose and Objectives

3.1 Hypothesis

Hypothesis A: Video-based PFMT can be easily followed and used to practice PFMT

Hypothesis B: Video-based PFMT will help to reduce symptoms of pelvic floor disorders and improve the QoL of participants

3.2 Primary Objective

The primary objective is to the determine the feasibility and accessibility of using a video-based PFMT for patients with pelvic floor dysfunction.

3.3 Secondary Objective (if applicable)

To determine whether video-based PFMT can improve symptomatology and QoL.

4 Study Design

4.1.1 General Design Description

Patients will be enrolled during the clinic visit when they are initially referred for PFR. It will be explained that they are forgoing standard of care of all in-person pelvic floor rehabilitation and will complete the initial survey at this time. They will then have an introductory session with a pelvic floor therapist to assess their baseline function, which does not differ from the standard of care other than the pelvic floor therapist using a graded scoring system to assess exercise completion ability. This initial visit will be prioritized as the waitlist for pelvic floor rehabilitation can be up to 6 months. Then they will complete the 8 weeks of remote, self-directed video-based PFMT. At the midway point after completing 4 weeks of the remote, self-directed video based PFMT, patients will be contacted by a member of the research team to elicit feedback and complete a system usability scale to evaluate patient perspective of the online training program. After this period, they will have another in-person session with the same pelvic floor physical therapist to assess ability to complete exercises successfully with the same graded scoring system. They will then complete a system usability scale survey and repeat the initial surveys for QoL and symptom surveys within 2 weeks of the final in person pelvic floor therapist appointment.

4.1.2 Study Date Range and Duration

Recruitment period: March 2025 – August 2026

4.1.3 Number of Study Sites

Yale New Haven Hospital and the associated clinic sites

4.2 Outcome Variables

4.2.1 Primary Outcome Variables

SUS, percentage of video completion, change in the graded evaluation of exercises by pelvic floor therapist pre and post intervention, adverse events

4.2.2 Secondary and Exploratory Outcome Variables (if applicable)

Secondary outcomes will include the Short Form-12 (SF-12), along with one of the four appropriate symptom-focused surveys: 1) Cleveland Clinic Incontinence Score (CCIS), 2) Patient Assessment of Constipation Symptoms (PAC-SYM), 3) International Consultation on Incontinence Questionnaire Short Form (ICIQ-SF), and 4) Pelvic Organ Prolapse Symptom Score (POP-SS). These surveys are attached.

4.3 Study Population

The study population will include patients who have an indication for referral to PFR.

4.3.1 Number of Participants

A total of 10-20 patients will be enrolled in the study.

4.3.2 Eligibility Criteria/Vulnerable Populations

Eligibility will be assessed by a member of the study team reviewing outpatient clinic visits to colorectal surgery and urogynecology clinics at Yale New Haven Hospital and its associated ancillary clinic sites.

In order to be eligible for inclusion in this study, an individual must meet all of the following criteria:

1. Diagnosis of one of the five following diagnoses with referral for PFR
    a. Urinary incontinence
    b. Pelvic pain
    c. Fecal incontinence
    d. Postpartum-related symptoms
    e. Constipation
2. 18 years or older
3. English-speaking
4. Access to internet

Vulnerable populations: none

Exclusion criteria:

1. Unable to access web-based videos
2. Unable to speak English

5 Study Methods/Procedures

5.1 Study Procedures

1. Initial survey completion at initial clinic visit
2. In person visit with pelvic floor physical therapist
3. 8 weeks of online video-based PFMT program
4. Midway check-in with research member and survey completion
5. Completion in person visit with pelvic floor therapist
6. Completion surveys after 8 weeks of video-based PFMT

5.1.1 Data Collection

The survey information will be collected by a trained survey either in person or filled out independently by the study participant using a Yale-licensed Qualtrics platform. If there are technical difficulties, a paper-based version of the assessment tool will be substituted and later inputted. This data and the clinical data will be collected from the electronic medical records (e.g., Epic® chart reviews) and will be securely stored in the HIPAA compliant RedCap database. The assessment by the pelvic floor therapist will also be secured inputted into the RedCap database.

At the completion of the study period, we will compare the assessment of PFMT adequacy as assess by the pelvic floor therapists, usability of the video-based application, and any adverse events as our primary outcome for feasibility and safety. We will analyze the change in the SF-12 score, CCIS, PAC-SYM, pre and post intervention to evaluate any change as our secondary outcome evaluating for efficacy of video-based PFMT.

5.1.2 Adverse Events Definition and Reporting

All patients will be provided with the contact information of the Principal Investigator if they feel that they have been adversely affected by participating in the surveys or in the in-person or video-based PFMT sessions.

This protocol presents minimal risks to the subjects and Unanticipated Problems Involving Risks to Subjects or Others (UPIRSOs), including adverse events, are not anticipated. In the unlikely event that such events occur, Reportable Events (which are events that are serious or life-threatening and unanticipated (or anticipated but occurring with a greater frequency than expected) and possibly, probably, or definitely related) or Unanticipated Problems Involving Risks to Subjects or Others that may require a temporary or permanent interruption of study activities will be reported immediately (if possible), followed by a written report within 5 calendar days of the Principal Investigator becoming aware of the event to the IRB (using the appropriate forms from the website) and any appropriate funding and regulatory agencies. The investigator will apprise fellow investigators and study personnel of all UPIRSOs and adverse events that occur during the conduct of this research project through weekly lab meetings to survey the progress of the study. The protocol's research monitor(s), e.g., Data and Safety Monitoring Boards, study sponsors, funding and regulatory agencies, and regulatory and decision-making bodies will be informed of physical harm adverse events within 5 days of the event becoming known to the principal investigator.

5.2 Study Schedule

Initial surveys completed at initial clinic visit

In person visit standard of care visit with pelvic floor therapist and graded scoring of exercise completion ability (1 hour)

8 weeks of online, video-based PFMT program; videos were created by Yale (website: https://pelvicfloortherapy.wixsite.com/yale-video-based-pel)

Midway check-in with research team and completion of SUS survey (15 minutes)

Completion in person standard of care visit with pelvic floor therapist and graded scoring of exercise completion ability (1 hour)

Completion surveys after 8 weeks of video-based PFMT (within 1 week of in person visit)

5.3 Informed Consent

Consent forms describing in detail the study intervention, study procedures, and risks are given to the participant and written documentation of informed consent is required prior to starting intervention/administering study intervention. The consent materials are submitted with this protocol:

- Consent form

5.3.1 Screening (if applicable)

Not applicable

5.3.2 Recruitment, Enrollment and Retention (if applicable)

Potential subjects will be identified by reviewing clinic patients with the provider and patients will be targeted for in-person recruitment by the treating provider with the trained research assistant present. We may contact patients of treating providers who have given written agreement to have their patients who are eligible for PFR to be contacted if unable to consent in person. The study will be introduced to the patients by the treating provider and the trained research assistant will consent and complete initial survey administration during the initial visit, or if not able will complete the survey via telephone call or electronic communication. Patients will be compensated a total of $50 for attendance of the first and last in-person PFMT sessions.

5.3.3 Study Visits (is applicable)

1. Initial clinic visit
    a. SF-12
    b. And one of the following surveys:
        i. CCIS
        ii. PAC-SYM
        iii. ICIQ-SF
        iv. POP-SS
2. In person visit with pelvic floor therapist (1 hour)
    a. Introduction to PFMT clinical visit by a certified pelvic floor therapist
    b. Graded evaluation of pelvic floor exercises on a scale of 1-3 (1= cannot replicate in any meaningful way, 2 = partially, 3 = fully perform)
3. Midway check-in with research team member (15 minutes)
    a. Elicit feedback on self-direct video-based training
    b. Completion of SUS
4. Completion in person visit clinical visit with pelvic floor therapist (1 hour)
    a. Graded evaluation of pelvic floor exercises on a scale of 1-3 (1= cannot replicate in any meaningful way, 2 = partially, 3 = fully perform)
5. Completion survey within 1 week of seeing pelvic floor therapist
    a. SF-12
    b. And one of the following surveys:
        i. CCIS
        ii. PAC-SYM
        iii. ICIQ-SF
        iv. POP-SS
    c. SUS

5.4 Statistical Method

5.4.1 Statistical Design

Demographic information will be reported using descriptive statistics. Categorical variables will be analyzed with chi-squared test and continuous variables will be analyze with Wilcoxon Rank sum test. Paired T-test will be used to evaluate differences pre and post treatment of the following measures:

1) amount of supervision and cues needed by the pelvic floor therapist
2) difference in graded scoring of exercises by the pelvic floor therapist
3) CCIS scores
4) PAC-SYM score
5) SF-12 Scores
6) ICIQ-SF Scores
7) POP-SS Scores

5.4.2 Sample Size Considerations

This is a feasibility study with no prior literature publishing on the use of video-based PFMT. Based on prior feasibility studies, the sample size will be between 10-20 patients.

5.4.3 Planned Analyses

The primary analysis to evaluate for feasibility and safety will involve the percentage of patients who successfully learned PFMT, analysis of incidence of adverse events and overall score of usability for the web-based videos. We will also evaluate the ability of participants to successfully complete the exercises based on the graded scoring by the pelvic floor therapists. Analysis of secondary outcomes

related to symptom improvement and quality of life will analyze SF-12, CCIS, PAC-SYM, ICIQ-SF and POP-SS scores.

5.4.4 Analysis of Subject Characteristics (if applicable)

We will analyze general demographic information such as age, sex, race, ethnicity.

5.4.5 Interim Analysis (if applicable)

5.4.6 Handling of Missing Data

Patients who complete less than 75% of the videos or do not attend both initial and final in person pelvic floor therapy visit will be excluded from the study. A complete case analysis will be performed for patients who have completed between 75-100% of the videos.

6 Trial Administration

6.1 Ethical Considerations: Informed Consent/Assent and HIPAA Authorization

Consent forms will be Institutional Review Board (IRB)-approved and the participant will be asked to read and review the document online. The consent form will be stored and accessible through RedCap. The trained research assistant will explain the research study to the participant and answer any questions that may arise. This conversation will take place in a private room.

Participants will have the opportunity to carefully review the electronic consent form and ask questions prior to signing. The participants should have the opportunity to discuss the study with their family or surrogates or think about it prior to agreeing to participate. The participant will sign the informed consent document prior to any procedures being done specifically for the study. Participants must be informed that participation is voluntary and that they may withdraw from the study at any time, without prejudice. A physical copy or emailed copy of the informed consent document will be given to the participants for their records. Patients will be compensated with $25 for each in-person visit with the pelvic floor therapist (initial and final) for a total of $50. The Informed Consent form is included as an attachment to this protocol.

6.2 Institutional Review Board (IRB) Review

This prospective feasibility protocol will be submitted to the IRB for review and approval. Approval of the protocol must be obtained before initiating any research activity. Any change to the protocol or study team will require an approved IRB amendment before implementation. The IRB will determine whether informed consent and HIPAA authorization are required.

A study closure report will be submitted to the IRB after all research activities have been completed.

Subject Confidentiality

Participant confidentiality and privacy is strictly held in trust by the participating investigators, their staff, and the sponsor(s) and their interventions. Therefore, the study protocol, documentation, data,

and all other information generated will be held in strict confidence. No information concerning the study or the data will be released to any unauthorized third party without prior written approval of the sponsor.

All research activities will be conducted in as private a setting as possible.
The study monitor, representatives of the Institutional Review Board (IRB), or regulatory agencies may inspect all documents and records required to be maintained by the investigator, including but not limited to, medical records (office, clinic, or hospital) for the participants in this study. The clinical study site will permit access to such records.

The study participant's contact information will be securely stored on HIPAA-compliant Yale REDCap Database for internal use during the study. At the end of the study, all records will continue to be kept in a secure location for as long a period as dictated by the reviewing IRB, Institutional policies, or, if applicable, sponsor requirements.

Study participant research data, which is for purposes of statistical analysis and scientific reporting, will be transmitted to and stored in Yale REDCap. This will not include the participant's contact or identifying information. Rather, individual participants and their research data will be identified by a unique study identification number. The study data entry and study management systems used will be secured and password protected. After participation in the study is complete, we may access patient records to collect demographic, medical history and surgical history information.

6.3 Deviations/Unanticipated Problems

A protocol deviation is any noncompliance with the study protocol. The noncompliance may be either on the part of the participant, the investigator, or the study site staff. As a result of deviations, corrective actions are to be developed by the site and implemented promptly.

It is the responsibility of the site investigator to identify and report deviations within 5 working days of identification of the protocol deviation. All deviations must be addressed in study source documents, reported to the study sponsor, and the reviewing Institutional Review Board (IRB) per their policies.

Unanticipated problems involving risks to participants or others include, in general, any incident, experience, or outcome that meets all of the following criteria:

- Unexpected in terms of nature, severity, or frequency given (a) the research procedures that are described in the protocol-related documents, such as the Institutional Review Board (IRB)-approved research protocol and informed consent document; and (b) the characteristics of the participant population being studied;
- Related or possibly related to participation in the research (“possibly related” means there is a reasonable possibility that the incident, experience, or outcome may have been caused by the procedures involved in the research); and
- Suggests that the research places participants or others at a greater risk of harm (including physical, psychological, economic, or social harm) than was previously known or recognized.

The investigator will report unanticipated problems (UPs) to the reviewing Institutional Review Board (IRB) and to the study sponsor. The UP report will include the following information:

- Protocol identifying information: protocol title and number, PI's name, and the IRB project number;
- A detailed description of the event, incident, experience, or outcome;
- An explanation of the basis for determining that the event, incident, experience, or outcome represents an UP;
- A description of any changes to the protocol or other corrective actions that have been taken or are proposed in response to the UP.

To satisfy the requirement for prompt reporting, UPs will be reported using the following timeline:

- UPs that are serious adverse events (SAEs) will be reported to the IRB and study sponsor, if applicable within 5 days in accordance with of the investigator becoming aware of the event.
- Any other UP will be reported to the IRB and study sponsor within [insert timeline in accordance with policy] of the investigator becoming aware of the problem.
- All UPs should be reported to appropriate institutional officials (as required by an institution's written reporting procedures), the supporting agency head (or designee), and the Office for Human Research Protections (OHRP) within 5 working days of the IRB's receipt of the report of the problem from the investigator.

6.4 Data Quality Assurance

To ensure high standards of data integrity, we will have one trained pelvic floor therapist assessing all our patients thus eliminating inter-rater variability and standardizing the method of assessing and collecting metrics of pelvic floor muscle therapy performance. In addition, there is going to be one researcher to consent and extract patient information. We are using validated assessment tools to evaluate for system usability and to evaluate secondary outcomes.

6.5 Study Records

Study records including consent forms, survey responses and data extracted from the medical record will be stored on the secure RedCap Database.

6.6 Access to Source

Data will be maintained per Medical Records policy in the password protected, secure, Health Insurance Portability and Accountability Act (HIPAA) compliant, web-based RedCap database with a built-in audit trail.

Only Institutional Review Board (IRB) approved research team members who have current HIPAA and Collaborative Institutional Training Initiative (CITI) Good Clinical Practice (GCP) and human subjects protection training will be authorized to access records.

6.7 Data or Specimen Storage/Security

All data will be maintained in RedCap database as described above.

6.8 Retention of Records

Data collection and integrity will be the responsibility of the submitting principal investigator and data-integrity will be confirmed and validated monthly.

6.9 Study Monitoring

The study records will be maintained until 3 years from initial enrollment after which point the database will be destroyed.

6.10 Study Modification

Any change to the protocol or study team will require an approved IRB amendment before implementation.

6.11 Study Completion

Recruitment will go from March 2025 with expected patient follow-up to last no longer than August 30 2026. Records will be retained for 3years from the end of the recruitment period with closure report submitted to the IRB on August 30th 2026.

6.12 Funding Source

This project will be funded from departmental start-up funds provided to the submitting principal investigator.

6.13 Conflict of Interest Policy

The independence of this study from any actual or perceived influence, such as by the pharmaceutical industry, is critical. Therefore, any actual conflict of interest of persons who have a role in the design, conduct, analysis, publication, or any aspect of this trial will be disclosed and managed. Furthermore, persons who have a perceived conflict of interest will be required to have such conflicts managed in a way that is appropriate to their participation in the trial. The study leadership in conjunction with the appropriate conflict of interest review committee has established policies and procedures for all study group members to disclose all conflicts of interest and will establish a mechanism for the management of all reported dualities of interest.

All investigators will follow the applicable conflict of interest policies.

6.14 Publication Plan

Data is tentatively planned to be presented at a national meeting focused on pelvic floor disorders with subsequent publication to affiliated journal. The primary investigator will hold the primary responsibility for publishing the study results.

Appendices

Appendix #	Title	Section	Topic

List of Tables
