# Supplementary material for: Video-based pelvic floor muscle therapy for patients with pelvic floor disorders: A Protocol for a prospective single-arm pilot and feasibility study
Source: PLoS One. 2025 Oct 24;20(10):e0329883. doi: 10.1371/journal.pone.0329883 (PMC12551819; doi:10.1371/journal.pone.0329883)
Supplement: S2 File — Weekly logs provided to study participants for self-reporting of weekly exercise frequency. (PDF) [file pone.0329883.s003.pdf]

Patient ID: ____

Weekly Log

Please check off each day that you practiced the pelvic floor exercises (you may click more than one day). As a reminder, the goal is to exercise for at **least 20 minutes** for **3 days a week.**

- Monday
- Tuesday
- Wednesday
- Thursday
- Friday
- Saturday
- Sunday

Please add any comments below (not required):

____
